# Perceptions of wellbeing and quality of life following participation in a community-based pre-operative exercise programme in men with newly diagnosed prostate cancer: A qualitative pilot study

**DOI:** 10.1371/journal.pone.0253018

**Published:** 2021-06-10

**Authors:** Lisa Loughney, Rachel McGowan, Kiaran O’Malley, Noel McCaffrey, Bróna Furlong, Deirdre Walsh

**Affiliations:** 1 School of Health and Human Performance, Dublin City University, Dublin, Ireland; 2 Royal College of Surgeons in Ireland, Dublin, Ireland; 3 School of Psychology, National University of Ireland Galway, Galway, Ireland; 4 Department of Urology Surgery, Mater Misericordiae University Hospital, Dublin, Ireland; 5 Department of Social Science, Athlone Institute of Technology, Athlone, Ireland; Edinburgh Napier University, UNITED KINGDOM

## Abstract

**Background:**

Men with a newly diagnosed prostate cancer are often treated by surgery. The time window between cancer diagnosis and surgery causes high levels of uncertainty and stress, which negatively impact quality of life (QoL). We previously reported a larger intervention pilot study which demonstrated that participation in a community-based pre-operative exercise programme significantly improved physical fitness and health-related quality of life in men with prostate cancer prior to surgery. The aim of the current pilot study was to get an insight into men’s perceptions of wellbeing and QoL following completion of the pre-operative exercise programme.

**Methods:**

From November 2017 to June 2018, men scheduled for prostate cancer surgery were recruited and took part in a prescribed community-based pre-operative exercise programme in the time available between referral and surgery. Following completion of the pre-operative exercise programme (within 1 week before surgery), participants took part in one semi-structured interview which explored four broad QoL domains: physical, psychological, social, and spiritual wellbeing. Data were analysed using thematic analysis (a bottom up/inductive analysis).

**Results:**

Eleven men were recruited: mean standard deviation (SD) age was 60 ± 7 years. Data supported four main themes. Participation in the community-based pre-operative exercise training programme (over a mean (SD) of 4 ± 2 weeks) provided participants with: 1) a teachable moment; 2) a journey of preparation; 3) a sense of optimism; and 4) social connectedness prior to surgery.

**Conclusion:**

This study provides an insight into how the exercise programme impacted wellbeing and QoL in men preparing for prostate cancer surgery. These findings highlight the important role that exercise prehabilitation plays for men preparing for prostate cancer surgery. Such exercise programmes can be easily implemented into standard cancer pathways by establishing relationships between hospital teams and community exercise programmes.

## Introduction

In Ireland, prostate cancer is the second most common cancer in men and the fourth most common worldwide [1, 2]. Twenty-eight percent of people with a newly diagnosed prostate cancer are treated by surgery and hormone therapy and 36% are treated by radiotherapy [3]. Treatment can have many adverse effects such as sexual dysfunction, urinary and bowel problems, fatigue, distress, anxiety and depression [4, 5]. For those treated by surgery, the time window between cancer diagnosis and surgery can cause high levels of uncertainty and stress, both of which can negatively effect quality of life (QoL). Psychological outcomes pre-operatively can impact post-operative outcomes both in the short- and long-term [6]. Furthermore, pre-operative intrusive thoughts can negatively impact QoL and that this can be linked to depressed mood and waking up with anxiety up to 2 years following radical prostatectomy [7, 8].

Physical activity can greatly enhance wellbeing and QoL across several domains for cancer survivors such as physical (i.e. managing the physical consequences of cancer and its treatment), psychological (i.e. evoking positive self-perceptions), social (i.e. feeling understood by others), and spiritual (i.e. redefining life purpose) [9]. Moreover, recent guidance and recommendations on exercise and cancer report the beneficial effects exercise has on improving survival following a prostate cancer diagnosis as well as its’ positive impact on fatigue, anxiety, depression, physical function and QoL [10]. There is a growing evidence-base of the important role prehabilitation has on optimising patient outcomes for surgery. However, studies on community-based exercise prehabilitation prior to prostatectomy is relatively new. One previous pre-operative exercise study demonstrated improvements in physical functioning in men with prostate cancer preparing for surgery [11] whilst another study, from our group, showed significant improvements on physical fitness, overall health-related quality of life (EQ-5D) and emotional functioning subscale (EORTC QLQ-C30) following a community-based pre-operative programme [12].

We previously reported a larger intervention pilot study which demonstrated that participation in a community-based pre-operative exercise programme significantly improved physical fitness and health-related quality of life in men with prostate cancer prior to surgery. The aim of the current pilot study was to get an insight into men’s perceptions of OoL domains for physical, psychological, social, and spiritual wellbeing following participation in a community-based pre-operative exercise programme prior to prostate cancer surgery.

## Materials and methods

**Fig 1:**
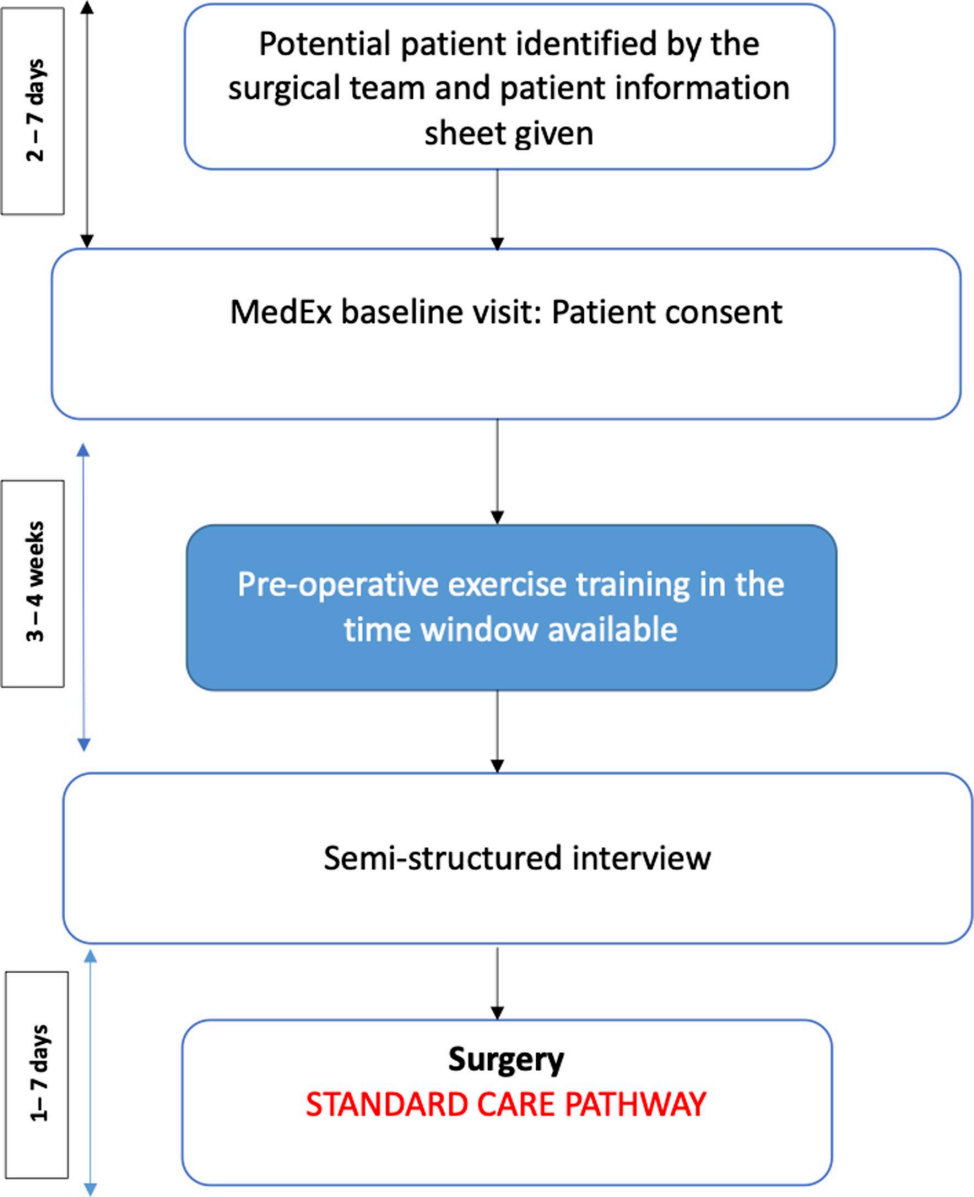
Study flow illustrating patient pathway.

This was a nested study of a larger interventional pilot study [12], approved by Dublin City University Research Ethics Committee (REC/2015/207). We recruited 11 consecutive participants with prostate cancer between November 2017 and June 2018 following referral from the Mater Misericordiae University Hospital (MMUH), Ireland. This study is reported according to the Consolidation Criteria for Reporting Qualitative Research (COREQ) [13]. The study flow diagram is presented in Fig 1.

### Participants

Inclusion criteria included men with prostate cancer aged ≥18 years who were scheduled for surgery. Exclusion criteria included contraindications to exercise including uncontrolled cardiovascular conditions, significant skeletal muscle, orthopaedic, or neurological condition, or cognitive decline, significant mental illness or intellectual disability that prevented participation in a physical training programme. Patient information leaflets were issued at the outpatient prostate cancer clinic and those interested made contact or were contacted by the lead study clinical exercise physiologist (LL).

### Procedures

A purposive sampling strategy was employed within this nested study. All participants were made aware of the research aim (i.e. to explore participants lived experiences/personal accounts of taking part in an exercise prehabilitation programme on their perception of wellbeing and QoL). Eleven participants were approached and agreed to participate in this study. Written consent was obtained at their baseline visit (prior to the starting the prehabilitation programme).

Semi-structured individual interviews were conducted in the MedEx centre at Dublin City University [14] following completion of the pre-operative exercise training programme (within 1 week prior to surgery). No baseline/pre-exercise intervention interviews were conducted. Semi-structured interviews were conducted by two female authors LL (Clinical Exercise Physiologist., BSc, MSc, PhD) & RMcG (MSc student undertaking data collection, BSc), both of whom were trained in research interviewing techniques by senior author (DW., Psychology., BSc, MSc, PhD). LL was lead study co-ordinator and exercise lead therefore she had the opportunity to establish relationships with participants whilst RMcG was not involved in the provision of the exercise prehabilitation programme and did not have an established relationship with participants prior to conducting interviews. LL has 10 years of experience in delivering and managing prehabilitation programmes in this area of research. RMcG has an interest in Health Psychology, specifically in terms of health behaviour change in clinical populations. Both authors had assumptions about the potential benefits of prehabilitation based on their previous experience and were interested to investigate prehabilitation within a different surgical oncological population (i.e., prostate cancer) [15–18]. Only one researcher was present for the interviews.

All interviews were audio-recorded in a quiet room. Semi-structured interviews were carried out using open-ended questions previously used by the Fit4Surgery group [19] which were adapted to suit the current study and explored four domains of wellbeing and QoL: social, spiritual, psychological and physical, which are in accordance with Ferrell et al., 1995 [20], as well as the participant experience of participation in the exercise programme. Each interview commenced by providing a brief introduction to the researcher and their background as well as an overview of the research aims (as per the participant information sheet). Participants were encouraged to lead the interview while general probing questions were used in order to follow up on different areas of interest. No field notes were taken during the interviews, however authors LL, DW and RMcG debriefed following each interview conducted. The interview guide and prompts used are presented in S1 Appendix.

### Community-based exercise intervention

The MedEx programme (now rebranded as ExWell Medical) is an established community-based, medically supervised, chronic illness rehabilitation programme that was based in the gym at Dublin City University campus [14]. The exercise intervention for this study was delivered as part of the MedEx programme. The exercise prescription is described elsewhere [12] and in Table 1 below using the FITT principle (Frequency, Intensity, Time, Type) and progression. In brief, participants started their pre-operative exercise programme following referral from the prostate cancer clinic at the MMUH and completed the programme in the time window available prior to surgery. Options for participation included undertaking either a centre-based exercise programme (CBEP) or a home-based exercise programme (HBEP) to facilitate equality of access to eligible participants. The HBEP catered for those living outside Dublin, working full time or for those that could not access the centre. The exercise intervention was delivered alongside the Cancer Prepare programme as part of the overall MedEx programme. Assessments for all participants took place at the same time as the Cancer Prepare service which included between 8–12 participants with different cancer types and at different stages of their cancer journey (pre/post-operatively). This provided participants with an opportunity to interact with those on the programme.

**Table 1:** Exercise training prescription described using the FITT principle (frequency, intensity, time, type) and progression.

**Frequency**	Participants were asked to complete 3–5 sessions per week depending on the time window before surgery (i.e. if surgery was scheduled 2 weeks following baseline assessment, participants were advised to complete 5 sessions per week, if surgery was scheduled >2 weeks following baseline assessment, participants were advised to complete ≥3 sessions per week).
**Intensity**	Intensity of aerobic exercise included: (1) Interval (moderate to high intensity) exercise training which included a combination of moderate and high intensities (RPE scale of 13: somewhat hard and 15: hard) [21]; (2) High intensity exercise training (RPE scale of 16) [22]. Resistance training included 3 sets x 12 repetitions. The weight was selected based on the participant ability (using a weight where they could complete 12 repetition maximum with a minimum 30 sec recovery period between sets).
**Time**	Time of the exercise session ranged between 40 to 60 mins. Interval: For the first interval exercise session, participants took part in a 30 minute session which incorporated 5 minute warm-up followed by 4 repeated bouts of moderate intensity (3 min) to high intensity (2 min) intervals followed by a 5 minute cool down. From the second session onwards, participants exercised for 40 minutes which incorporated a 5 minute warm-up, six repeated bouts of moderate intensity (3 min) to high intensity (2 min) intervals followed by a 5 minute cool down. High: For the high intensity exercise session, participants took part in 17.5 minutes of exercise which incorporated a 2 minute warm-up, 5 repeated bouts of high intensity (1 min) and recovery (90 seconds) intervals followed by a 3 minute cool down”. Resistance training: 3 sets x 12 repetitions (approx‥ 20 minutes).
**Type**	The CBEP aerobic exercise modalities included upright cycle ergometer; recumbent cycle ergometer; treadmill; elliptical ergometer; and rowing ergometer, depending on patient preference. The CBEP resistance training involved a circuit of strength 8–10 stations alternating upper and lower body exercises using the following machines: shoulder press; lateral pulldown; tricep press; squat; chest press; leg extension; hamstring curl; and back row. The HBEP aerobic exercise included walking; cycling; swimming; or any other aerobic activity they enjoyed. The HBEP resistance training involved upper and lower body exercises as per CPEB except using free weights/dumb bells dependent on availability.
**Progression**	Exercise intensity progressed every 5 sessions (if capable using the RPE scale as a guide). Resistance training progressed following comfortably completing three consecutive sessions.

Abbreviations: RPE (rate of perceived exertion); CBEP (centre-based exercise programme); HBEP (home-based exercise programme).

### Data analysis

Data were analysed from a pragmatic perspective [23] which embraces the use of mixed methods. This pragmatic approach generally agrees that all knowledge in this world is socially constructed, but that some individual experiences may be more widely held or recognisable than others [24]. This approach therefore aligned to the conduct of the current study whereby this qualitative pilot study was a nested study of a larger intervention pilot study [12] which investigated the compliance, adherence and efficacy of the exercise prehabilitation programme in improving fitness for surgery.

Semi-structured interviews were transcribed verbatim and checked for accuracy through multiple listenings within the thematic analysis process. Author RMcG transcribed and analysed all interviews. Transcripts and subsequent analyses were not returned to participants for comments/corrections. All participants who were partaking in this specific prehabilitation programme were invited to participant in this study. Eleven of the 11 (100%) eligible participants agreed to take part. The current group of participants were the only potential participants who could be sampled to partake in this study.

A thematic analysis was used to analyse the data using the six steps as described by Braun and Clark [25]. An inductive thematic analysis was conducted and themes were derived from the data. The following six phases of thematic analysis were conducted, while integrating best quality practices in relation to core elements of the Yardley framework for quality in qualitative health research [26]. Throughout the analysis phases, there was continued collaboration between LL, RMcG and DW in order to support this quality framework.

The six phases of thematic analysis and core areas of related quality assurance are discussed below. Phase one involved repeated listenings and readings of the transcripts in order to become familiar with the data and to gain a deeper understanding. Transcripts were checked against audio files and re-read with analytical notes. In the second phase, coding took place which involved actively reading the data line by line, taking note of any relevant patterns or meanings within the data and was conducted primarily by author RMcG (not involved in exercise programme delivery). This was a quality measure to ensure transparency of the initial coding. Following initial coding, in order to identify any significant ideas or patterns which were thought to be important or relevant, RMcG and DW (both independent to the exercise programme), discussed the initial codes of all transcripts developed by RMcG while being sensitive to the context in which this research was conducted. Within the third phase, the generation of themes (including sub-themes) occurred which involved synthesising the codes to accurately reflect the relevant over-arching themes within the data. A concept map of the interview data was created and discussed within the research team. For the fourth phase of the analysis, authors RMcG and DW reviewed each theme, sub-theme and the relationships between them and reviewed relevant codes and illustrative quotations to ensure coherency to the main scope of the relevant theme. The concept map of Phase four of the thematic analysis is presented in S2 Appendix. This concept map was used by authors RMcG and DW as a basis for discussions and refinement of the themes brought forward into Phase five. RMcG and DW discussed each theme and its validity and the value to the overall research question (i.e., exploring the experience of participation in a community-based pre-operative exercise programme, in the time between cancer diagnosis and surgery, and the subsequent impact on perceived wellbeing). Following these discussions and additional review of data, some sub-themes were combined with other relevant sub-themes.

Phase five involved naming the identified core themes using plain, explanatory language in an effort to capture the theme fully and accurately. Finally, a report was written up to provide a clear account of the analysis capturing the experience of perceptions of QoL and well-being following a pre-operative exercise programme in men with newly diagnosed prostate cancer. All authors compared, contrasted and discussed these to ensure they were relevant to the research question. To support the validity of the findings, supporting empirical evidence relevant to the research question was provided. This final phase of thematic analysis focused on aspects of quality assurance for qualitative research related to the ‘impact’ and ‘importance’ of the research. This element of quality is a crucial factor within this pilot study as the results are directly related to future study planning and design within this programme of research. The area of prehabilitation programmes in men with newly diagnosed prostate cancer is also relatively new and therefore, this study is an important step in building evidence relating to how such programmes impact men’s QoL and well-being.

## Results

From November 2017 to June 2018, 11 men were recruited to this nested study (note: nine/11 were part of the larger pre-post intervention study which included 15 men) [12]. Baseline characteristics are shown in Table 2. Mean (standard deviation (SD) age was 60 ± 7 years. Mean (SD) number of days between referral and starting the MedEx exercise programme was 4 ± 5 days and duration of pre-operative exercise training was 4 ± 2 weeks. Seven completed the CBEP, three the HBEP and one a combination of both. Adherence rates to the CBEP and HBEP (which was self-reported) was 84% and 100%, respectively.

**Table 2:** Participant characteristics.

	Prostate cancer participants (n = 11)
**Age (years)**	60 (7)
**Height (cm)**	176.2 (5.5)
**Weight (kg)**	92 (9.6)
**BMI (kg/m2)**	29.5 (2.7)
**No. of participants with co-morbidity** ^#^	7 (64)
**No. of participants taking medication** ^#^	6 (55)

Data are reported as mean (SD).

^**#**^Frequencies with percentages in parentheses.

**Fig 2:**
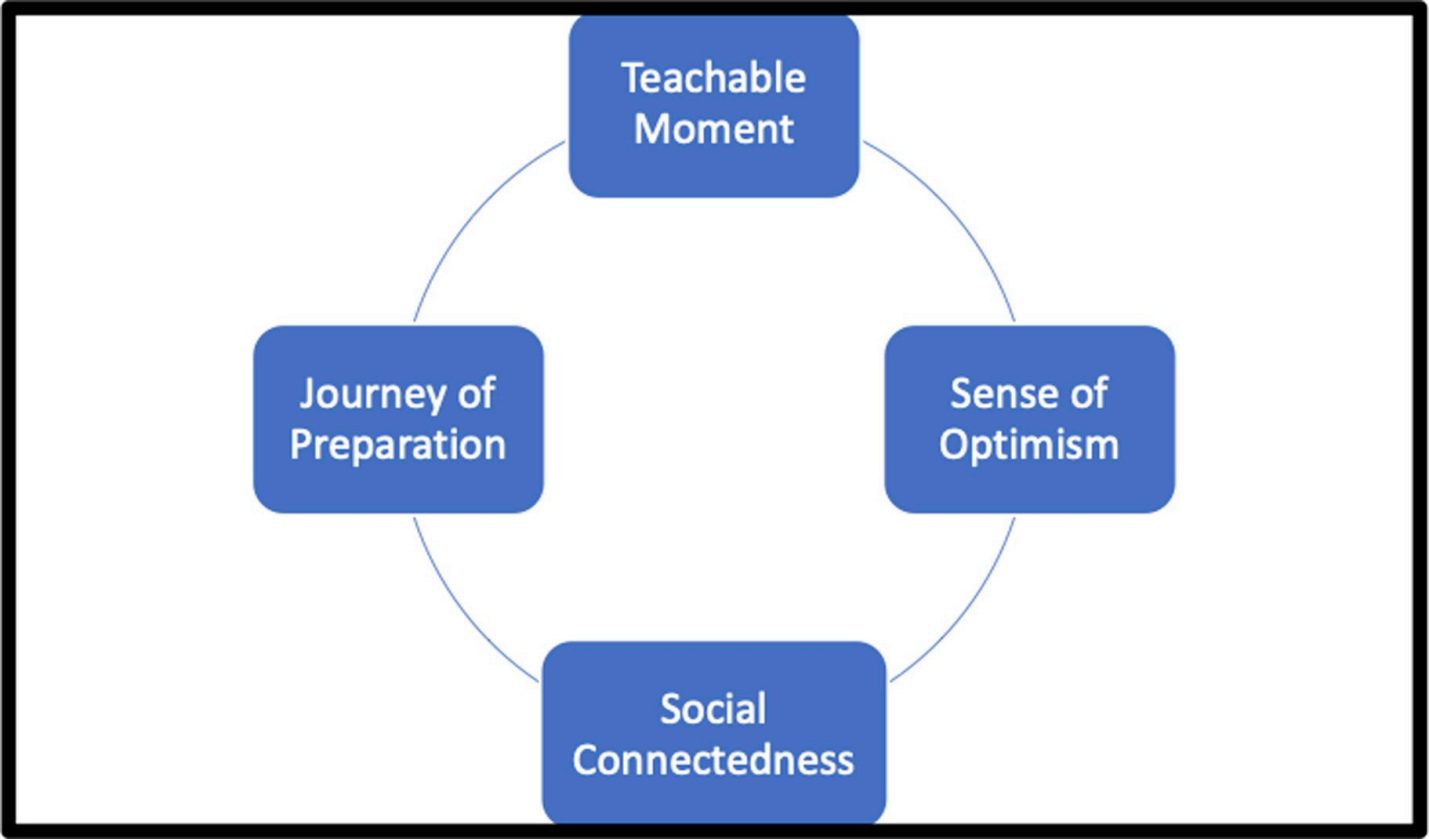
Illustration of main themes.

The mean (SD) duration of the 11 interviews was 11.5 min 22.7 sec ± 5 min 15 sec. Four main themes were supported by the data. Findings showed that engagement in the pre-operative exercise programme, both the CBEP and the HBEP, provided participants with: (1) a teachable moment; (2) journey of preparation; (3) a sense of optimism; and (4) a social connectedness which are reflected in the four main themes illustrated in Fig 2. There were no differences between the men who completed the CBEP and the HBEP. Of the three that completed the HBEP, two lived approximately twohour drive away from the centre and the other was a full-time business man, therefore the HBEP suited all their needs.

### (1) Teachable moment

Engagement in the programme following prostate cancer diagnosis acted as a teachable moment for the participants. It provided tangible components to focus on which led to greater health awareness and the tools to make positive health changes. Participants expressed positive changes from participating in the pre-operative exercise programme in a range of health behaviours including exercise, diet, alcohol consumption and lifestyle choices.

*“when I came here [to the exercise programme] first–blood pressure was way up…weight was way up… probably I would’ve had other problems if I hadn’t of come here*, *you know? And it has changed my life…*. *as regards fitness*, *and as regards what I eat as well*, *like*, *you know‥ I was never particular about that‥ I actually haven’t felt so well for a long time…*. *continuing on the way I was*, *probably four or five years down the road I would be really slow… it [the exercise programme] actually has improved my quality of life now and I think it will in the future as I know what to do now*. *I was probably a bit careless with myself*, *you know”-P6*

Many of the participants developed a more holistic view to health and gained a greater awareness of the value of health. The pre-operative exercise programme acted as a catalyst for change as many of them appeared to be hopeful for their future health.

The participants said that the exercise programme was a “trigger” to make changes in order to reach an optimal level of health.

*“I mean all it [the exercise programme] has done is kind of reinforce that I should never have given up the sport that I used to play which is Gaelic football and get back at it as quickly as possible*, *and try and build now*, *I’ll be 60 in a year and a half*, *so kind of set a target to get back to a level where I was in my forties … all my life I’ve played sports since I was a child*, *I was playing senior rugby when I was U-14*, *you know … so*, *I mean I’ve done sport*, *and even when I gave up sport*, *I liked refereeing and things like that*, *I coached kids and always had that interest*. *But like everyone else*, *life takes over*, *but it’s [the exercise programme] something now I actually will have to make time for*. *It’s kind of a little trigger that’s gone off again” -P2*

Participants expressed concern about their age in relation to the increased need to be more health conscious. The exercise programme reminded them to make more positive health changes in life:

*“I’d be very conscious that it [the exercise programme] would help me through the surgery of course*, *but… your health in general*, *just heightened awareness*, *just how important it is*, *particularly in the age profile I am, that* *health is key*.*”-P9*

Interactions with the staff made participants aware of other health benefits exercise offered which provided them with knowledge to make positive changes in the future:

“*You [staff] didn’t say I was going to be insulin free*, *or that*, *but you said it’s (type 2 diabetes)*, *it’s reversible with a certain amount of exercise*. *So that*, *to me*, *has given me a goal to run for*. *And I happened to be listening yesterday to the TV and a doctor said it was reversible*. *I’m not in that place at the minute to do that*, *but please God when I get over the surgery*, *ah*, *I’ll focus on that type of thing going forward*, *to try and get off the insulin”–P11*

*I am more aware from talking to you [staff]*, *‘cause I didn’t realise you could reduce your blood pressure by losing weight and… you know*? *So*, *it’s been good that way”–*P6

### (2) Journey of preparation

Many of the participants described the pre-operative exercise programme as a stepping stone on the road to ultimate recovery. There was a notable awareness of the physical health benefits of exercise and how this contributed to psychological preparedness for surgery:

*“Well I think it helps you to get over that bridge fast that’s why I’m doing it [the exercise programme]*. *If you’re fit and you’re in for an operation and you’re fairly fit which I would be… I think it eh… whatever keeps the mindset that I’m well able for this and that I can do it”- P9*

For some participants, the pre-operative exercise programme kept them “busy” giving them something to focus on and prepare for in the lead up to surgery:

*“yeah it’s kind of kept me*, *kept my brain busy as well*, *so I’m not thinking about the future and what I have to deal with*, *and more dealing with at present and trying to get myself ready for the op”*.*–P7*

Whilst other participants described the exercise programme as a mechanism to manage stress throughout their cancer journey and prepare themselves psychologically. The participants portrayed exercise as a mode of release to escape the difficulties they are faced with:

*“It’s given myself my own bit of time ehm and maybe you can think of things when you’re exercising but you can also when you’re exercising deal with any frustrations that you may have or any difficulties you may have because you can put it out through exercise”*-*P10*

### (3) Sense of optimism

Participation in the pre-operative exercise programme cultivated a positive attitude. This sense of optimism was a feature of the participants’ psychological wellbeing which seemed to be enhanced through participation in the programme. The participants seemed to understand the value of fostering and maintaining a sense of positivity during their preparation for surgery which was beneficial to their QoL:

*“I think that exercise has been the most positive part of all this*. *At the beginning I felt hopeless*. *But coming to the programme*, *I feel without MedEx I wouldn’t be as far as I am psychologically or holistically”-P1*

*“yeah it [the exercise programme] has benefited me psychologically*, *because like at the beginning I was like*, *oh why me? You know…*. *but there is people with worse cancers and worse off than me*, *so I think*, *you know*, *I can do this*, *I’ll get over this and I’ll move on with my life”-P2*

Participation in the pre-operative exercise programme provided an overall greater appreciation for the importance of maintaining and optimising one’s psychological health.

*“getting the prostate cancer sort of makes you think eh where you’re going in your life and how did you get it now I’m not saying any particular issue caused it but certainly a little bit more exercise maybe a little bit better diet… a little better various things in life… I also find eh I cope with things better now em because my mind is more alert and eh so eh yeh there the things that have really changed for me”*- *P8*

### (4) Social connectedness

Participants gained great value from people sharing their stories and experiences of the cancer journey with other participants. A strong sense of comradery and solidarity was fostered amongst participants through their shared experience. Participants valued practical advice from staff and support in relation to their pre-operative journey from peers with their lived cancer experience. This theme illustrates the value and usefulness of peer learning and sharing of experience through social connection rather than focusing solely on health care professional advice, research and ‘academic’ information:

*“people [fellow participants] are so willing to share with you their stories*, *and tell you*, *you know that’s not good*, *this is good*. *Like I’d be a big believer in research but it’s absolutely like having a practical on top of an academic*, *so that really is very useful”-P1*

For the participants, the friendly and patient-centred atmosphere at the CBEP contributed to the sense of wellbeing and particularly their sense of social integration. Interestingly, the social group within the exercise programme seemed to provide motivation within the participants ‘journey of preparation’ and highlight personal ‘teachable moments’;

*“meeting other people with similar experiences and it’s good to talk to them and see how well they are and how much they are enjoying the fitness [from taking part in the programme]”*- *P9*

Participants who engaged in the HBEP did not report lower levels of social connectedness in comparison to participants within the CBEP. The remote support from staff was also a valued part of the HBEP programme delivery:

*I have to be honest*, *you’re [staff] very very good*, *with the calls and anytime I sent an email*, *you replied to it*, *so to me it’s just keeping in touch*. *More than anything”- P11*

## Discussion

This study provides an insight into how exercise prehabilitation impacts wellbeing and QoL in men preparing for prostate cancer surgery with four key themes identified; a teachable moment, a journey of preparation, provided them with a sense of optimism and social connectedness in lead up to surgery.

Engagement with a pre-operative exercise programme following prostate cancer diagnosis provided participants with a concept often described as the “teachable moment”, i.e., an increased desire, willingness or capacity for change, which influences long term health and wellbeing [27]. The exercise programme fostered a holistic concept of health i.e., gave rise to greater health awareness and appeared to be a trigger to make changes in order to reach an optimal level of health. The teachable moment has been reported to be an important concept to promote health and well-being amongst a surgical population [28]. Interestingly, participants expressed interest to continue their exercise programme post-surgery. They noted positive impacts on physical and psychological health in the short time window between cancer diagnosis and surgery which gave them a confidence in their abilities to sustain the exercise habit beyond cancer surgery. A systematic review demonstrated that interventions can be an effective way of increasing physical activity at least 3 months following completion of an exercise programme [29]. Although little has been reported in newly diagnosed prostate cancer, the “teachable moment” has been described as a promising approach for clinicians to discuss behaviour change with patients efficiently and effectively in primary care [30]. It is interesting to note that in the current study, the consultant surgeon initiated the conversation about the exercise programme at cancer diagnosis which may have positively influenced participants’ health behaviours.

The pre-operative exercise programme acted as a stepping stone in the journey of preparation towards the crucial step of surgery in their road to recovery. It also provided participants with a sense of purpose and optimism. The overall feedback from participants was that they really enjoyed the programme in the lead up to their surgery. Attitudinal factors, particularly self-efficacy, a positive outlook and patient-perceived control are linked to improved functional recovery outcomes [31]. Physical activity has been shown to be a promising strategy for cancer survivors as it provides an opportunity to focus on health rather than illness [9]. A previous study in rectal cancer demonstrated that participation in a pre-operative exercise programme (hospital-based) cultivated positive attitudes for patients [19]. This initial pilot study supports that the exercise programme encouraged participants to adopt a proactive approach while awaiting surgery, which in turn, potentially enhanced perceived wellbeing.

The sense of comradery and solidarity that was fostered amongst participants during the programme acted as an avenue of social support and was identified as one of the key contributors to the participants’ wellbeing. This social connection with peers at MedEx facilitated sharing of individual lived experience of the pre-operative journey beyond ‘academic’ or ‘research’ information. Social networks and relationships as well as the social environment have been highlighted as being important aspects of the cancer care journey [32, 33]. In the current study, sharing stories about the cancer journey fostered a sense of connectedness amongst participants (note: participants on the HBEP attended MedEx at the same time as the CBEP and were integrated within the group). Similar effects have been reported in a study among women with breast cancer facing similar challenges where the importance of mutual understanding and support was documented [34, 35] and in a study with rectal cancer whereby participation in the exercise programme enhanced social connections [19]. Specific to men with prostate cancer, exercise interventions have been shown to reduce both psychological and social health problems as they combat the emasculating effects of prostate cancer treatment and they provide an element of social support [36]. It is possible that the social aspect of the exercise programme led participants to adopt a supportive social network which may have contributed positively to their perceived social wellbeing. Our study findings highlight the importance of relationships, social support and networking with peers among men with newly diagnosed prostate cancer while awaiting surgery. This finding requires further exploration in future research to understand the elements of a community-based intervention which facilitate social connection.

The important role of exercise in the cancer care pathway has been recently highlighted with published recommendations that exercise should be embedded as part of standard cancer care as it helps counteract the adverse effects of cancer and cancer treatment [37]. Pre-operative clinical guidelines and recommendations on exercise training have also been reported which provide practical guidance for providing safe and effective exercise [38]. In 2019, the American College of Sports Medicine also published guidance and recommendations for exercise and cancer [10]. Specific to Ireland, however, it has been recently reported that healthcare professionals need significant education in improving their knowledge on physical activity programmes in cancer [39]. Findings from the larger pre-post intervention pilot study [12] demonstrate that community-based exercise programmes such as the MedEx programme are effective in improving health-related components of fitness and QoL. The current study provides an insight into men’s perception of QoL and wellbeing prior to prostate cancer surgery following participation in the pre-operative exercise programme. Pre-operative exercise programmes can help to bridge the gap between the lack of specialist knowledge from healthcare professionals regarding cancer care and exercise by providing the knowledge and skills that can aid people with cancer to live more active and healthier lives and improve overall QoL and wellbeing.

### Strengths and weaknesses of this study

Strengths of this work include the novelty of the community-based model which is a more accessible, scalable and sustainable alternative to hospital-based programmes. Additionally, the rapid referral and access from the local hospital to the community allows for an immediate start without any delays which is important as the time window between decision to operate and surgery date can be short. The exercise programme was delivered alongside standard routine care in the time between diagnosis and surgery and the inclusion of a homogenous cancer type and treatment pathway which allows for findings to be generalisable.

Weaknesses include that the dose-response relationship (i.e. the no. of sessions participants completed as some may have had more/less time to derive affect from the programme) was not explored, which may have had negative implications for the results. The semi-structured interviews were carried out by research co-ordinator who was also leading the exercise programme (LL), although a number of randomly chosen interviews were also conducted by a research team member who was not directly involved in the exercise sessions (RMcG) in an effort to limit potential bias within interviews. It is noteworthy, that interviews conducted by both LL contained no clear differentiating positive bias towards the exercise programme in comparison to those conducted by RMcG. However, all participants responded positively to the exercise programme.

## Conclusion

This study provides an insight into how exercise prehabilitation impacts wellbeing and QoL in men preparing for prostate cancer surgery. These four main themes give valuable insight into the complex demands of the pre-operative journey and the positive role that exercise prehabilitation has on wellbeing and QoL in men with prostate cancer. Such exercise programmes can be easily implemented into standard cancer pathways by establishing relationships between hospital teams and community exercise programmes.

## Supporting information

S1 Appendix Semi-structured interview script. (DOCX)

S2 Appendix The concept map of phase four of the thematic analysis. (DOCX)
