# Supplementary material for: Perceptions of wellbeing and quality of life following participation in a community-based pre-operative exercise programme in men with newly diagnosed prostate cancer: A qualitative pilot study
Source: PLoS One. 2021 Jun 10;16(6):e0253018. doi: 10.1371/journal.pone.0253018 (PMC8191992; doi:10.1371/journal.pone.0253018)
Supplement: S1 Appendix — (DOCX) [file pone.0253018.s001.docx]

**Appendix 1: Semi-Structured Interview Script**

1. Can you describe your overall thoughts and perceptions of the exercise training program?
2. Has the experience of participating in the exercise program changed you in any way? If yes, please explain.
3. What does quality of life mean to you?
4. How would you describe your quality of life since your participation in the exercise program?
   1. Has your participation in the program impacted on your physical well-being (i.e.,  fatigue, pain levels, mobility)? If so, please explain.
   2. psychological well-being (i.e., sense of control)?
   3. social well-being (i.e., social interactions)?
   4. spiritual well-being (i.e., maintaining hope and deriving meaning)?
5. Can you tell me about your reasons for being interested in the exercise program?
6. Did you feel capable of performing the exercise program? If yes, please explain.
7. Did you have any concerns regarding your participation in the exercise training program? If yes, please explain.
8. Would you make any changes to the exercise program? If yes, explain.
9. Would you once again participate in the program? Why/why not?
10. Do you have any ideas or proposals for program improvements? If yes, explain.
11. Describe your thoughts and feelings relating to your upcoming surgery?
12. What does exercise mean to you at each stage of the cancer pathway?
