# Supplementary material for: Perceptions of wellbeing and quality of life following participation in a community-based pre-operative exercise programme in men with newly diagnosed prostate cancer: A qualitative pilot study
Source: PLoS One. 2021 Jun 10;16(6):e0253018. doi: 10.1371/journal.pone.0253018 (PMC8191992; doi:10.1371/journal.pone.0253018)
Supplement: Supplementary file 2 [file pone.0253018.s002.docx]

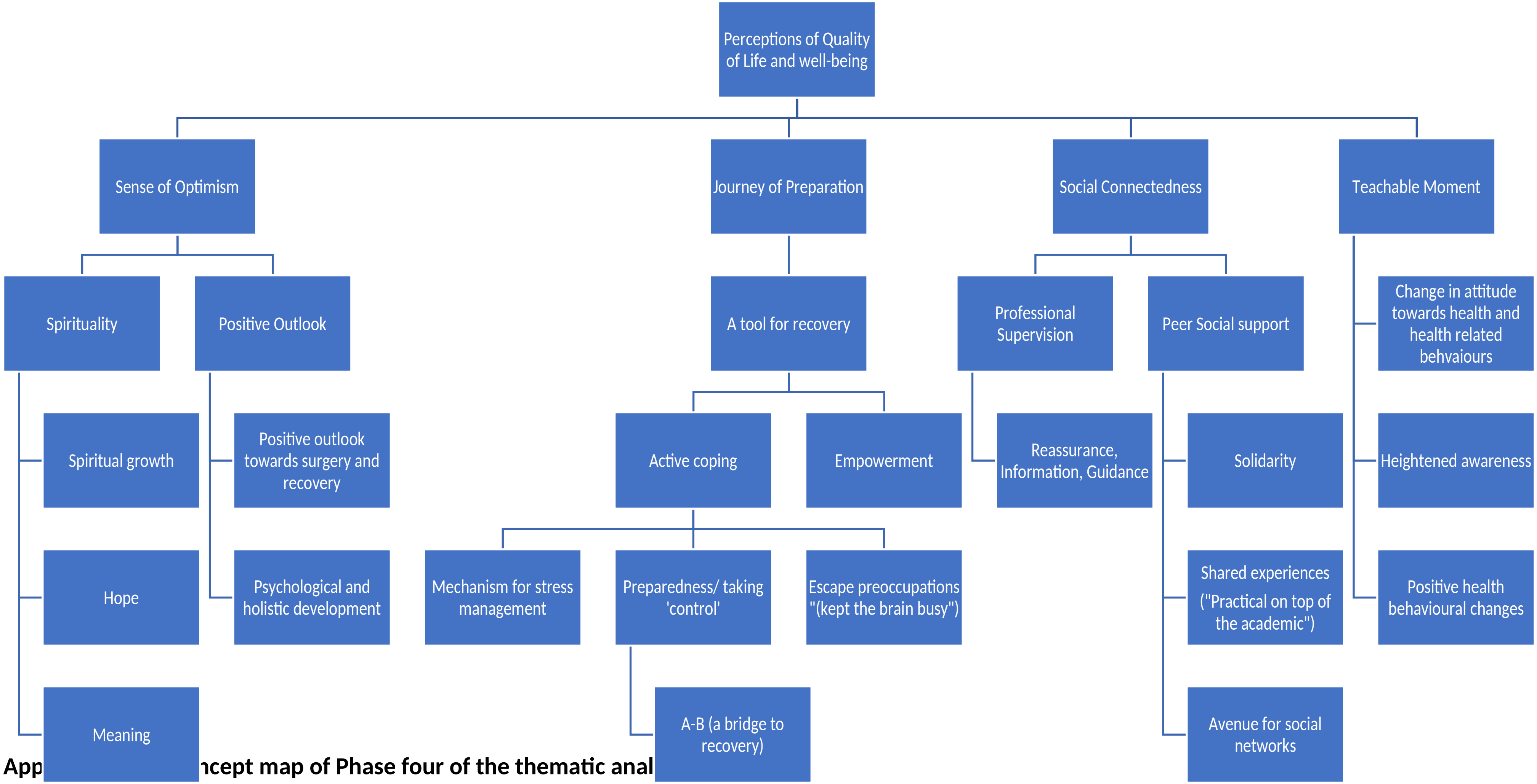

App[illegible]ncept map of Phase four of the thematic anal[illegible]
